# Supplementary material for: Hyperpolarized water through dissolution dynamic nuclear polarization with UV-generated radicals
Source: Commun Chem. 2020 May 8;3:57. doi: 10.1038/s42004-020-0301-6 (PMC9814647; doi:10.1038/s42004-020-0301-6)
Supplement: Supplementary file 2 — Supplementary Information [file 42004_2020_301_MOESM2_ESM.pdf]

Hyperpolarized water through dissolution dynamic nuclear polarization with UV-generated radicals

Arthur C. Pinon et al.

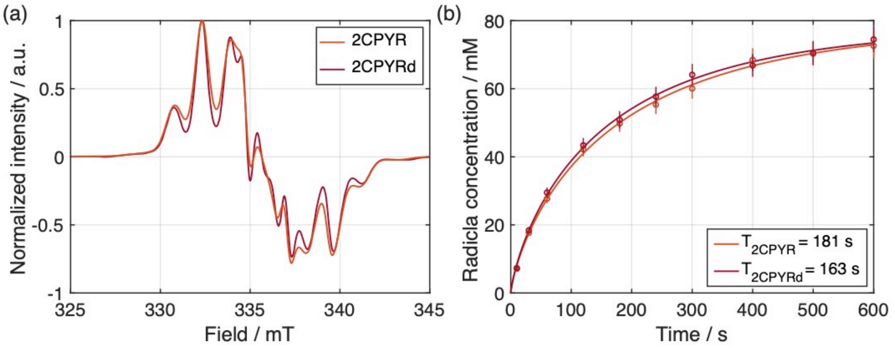

Supplementary Figure 1. UV-radical generation. (**a**) Normalized X-band ESR spectra after 10 min UV-light irradiation at 77 K and (**b**) radical generation time evolution of 8 frozen beads of 2CPYR_sample (orange) and 2CPYRd_sample (red). Data points and error bars are the average and standard deviation of repeated measurements from distinct samples (n = 3), respectively. The orange and red curves were obtained by fitting the data to a mono-exponential function. The time constant resulting from the fits are reported in the inset. Error on fits was below 5%.

Fits of the LOD-ESR spectra measured at 6.7 T and 1.15 K were obtained using the MATLAB-based software EASYSPIN.[1] Because of the random distributions of the radicals inside the glassing matrix, the PEPPER routine for powder spectra was employed. The fixed magnetic field/frequency swept mode was used. The exact magnetic field (i.e. 6707.2 mT) was determined from the dDNP polarizer ^{13}C Larmor Frequency (i.e. 71.800 MHz). A spin system S = ½ was assumed in all cases. The A-tensor for UV-irradiated PYR, 2CPYR and TEMPOL in a frozen glassing solvent has been carefully determined at X-band previously.[2,3] For each sample we kept the A-tensor as a constant of the fit and left the g-tensor as free parameters only, as it is more sensitive to the experimental and sample environment. Principal values of g-tensor and A-tensor are reported below and results of the fits in Supplementary Figure 2. The RMSD of the fit was 0.165, 0.402, and 0.225 for PYR, 2CPYR, and TEMPOL respectively.

PYR_sample: g-tensor = [2.00459 2.00464 2.00290];A-tensor(^{1}H) = [48 48 48] MHz (3 methyl group protons). *2CPYR*_sample: g-tensor = [2.00459 2.00464 2.00290];A-tensor(^{1}H) = [30 30 48] MHz (3 methyl group protons);A-tensor(^{13}C) = [40 40 157] MHz (c2-carbon). *TEMPOL_sample*: g-tensor = [2.00790 2.00595 2.00370]; A-tensor(^{14}N) = [20.48 17.68 101] MHz.

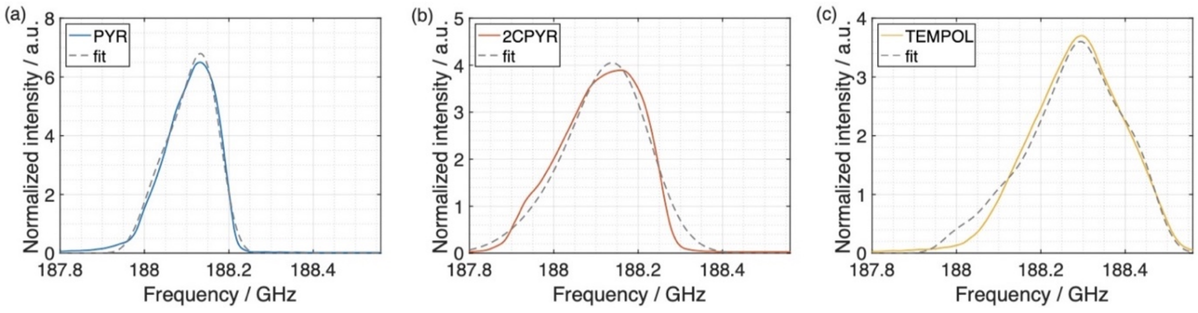

Supplementary Figure 2. LOD-ESR fits. The LOD-ESR spectrum fit in the frequency domain is reported for the PYR_sample (panel **a**), 2CPYR_sample (panel **b**) and TEMPOL_sample (panel **c**). Experimental data, acquired and measured at 6.7 T and 1.15 K, are shown as colored curve; calculated results are reported as dashed grey line. The area below the curve was normalized to unity according to $\int_{-\infty}^{\infty} g(\omega)d\omega = 1$, where $g(\omega)$ is the radical spectral function.

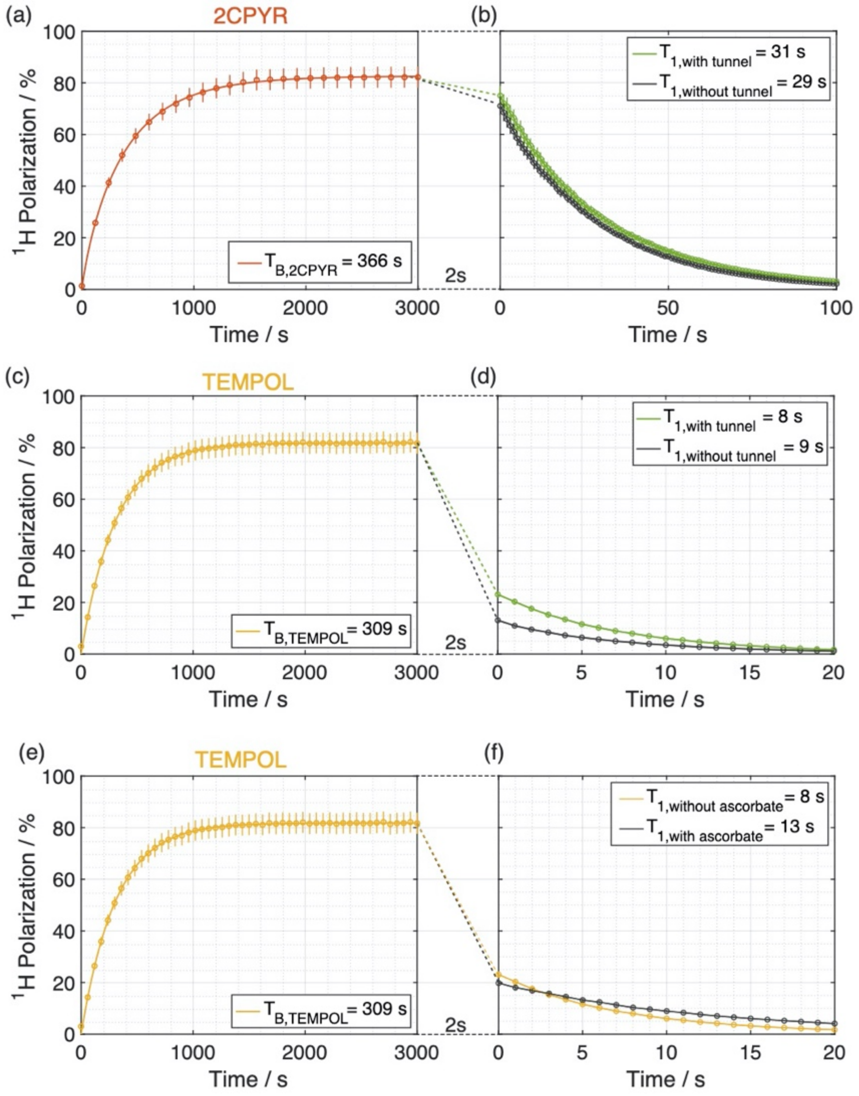

Supplementary Figure 3. Effect of the tunnel. The solid-state polarization at 6.7 T and 1.15 K is reported for the 2CPYR_sample (panel **a**) and the TEMPOL_sample (panel **c** and **e**). Upon achievement of maximum enhancement dissolution and direct transfer with (green dashed line) and without tunnel (grey dashed line) was performed, together with a dissolution with and without sodium ascorbate. Corresponding results for the liquid-state relaxation (color code) of same samples is shown in panel **b**, **d** and **f**, respectively. Data points and error bars are the average and standard deviation of repeated measurements from distinct samples (n = 3), respectively. All curves were obtained by fitting the data to a mono-exponential function. The different time constants resulting from the fits are reported in the insets. Error on fits was below 5%.

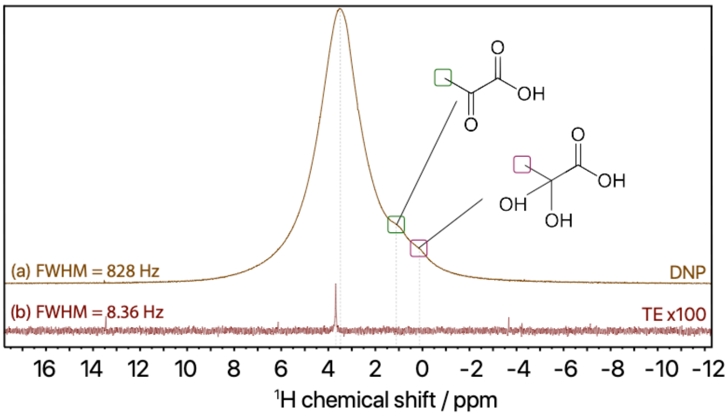

Supplementary Figure 4. Liquid-state NMR spectra. 2CPYR_sample hyperpolarized (**a**) and thermal equilibrium (**b**) spectrum measured after dissolution and DT transferred to a 10 mm NMR probe kept at 40 °C inside a 9.4 T NMR magnet. The resonances at 0.9 ppm and 0.1 ppm correspond to the methyl protons of the pyruvic acid and its hydrate form, respectively. In this specific experiment, the DNP enhancement calculated 22,953, corresponding to a polarization level of 70.4 %. Full width at half maximum (FWHM) are reported for the two spectra. An intense radiation dumping was recorded for the hyperpolarized signal.

Supplementary Methods

Radiation damping and polarization calculation

As described by Krishnen *et al.*,[4] radiation damping is when the precessing transverse magnetization of the nuclei after a rf pulse induces an electromagnetic field in the receiver coil. This creates an oscillating current that generates a transverse magnetic field at the same frequency. This induced field rotates the magnetization of the spins to its equilibrium toward the direction of the applied magnetic field, before other relaxation mechanisms can take effect. The rate at which the solvent magnetization is rotated back to equilibrium is given by a characteristic time constant known as the radiation damping time (τ_{rd}). Consequently, radiation damping will affect the measured polarization if the receiver is opened after the magnetization has already significantly rotated back, i.e if the radiation damping time is smaller than the spectrometer dead time. A sample of water polarized at thermal equilibrium in an NMR spectrometer operating at 400 MHz has a τ_{rd} value of around 20 ms.[4] In the high proton concentration experiment, the polarization is approx. 18,600 higher than thermal equilibrium (P(^{1}H) = 57%), measured with a pulse of 1°, and a proton concentration of 6.2 M which, assuming a similar coil quality factor as in ref 4, gives a radiation damping time constant of $\tau_{rd} = \frac{20}{18600} \cdot \frac{\sin(90°)}{\sin(1°)} \cdot \frac{110}{6.2} = 1.1$ ms. Consequently, with a dead time of 35 μs, the error on the magnetization calculation due to radiation damping is $1\text{-}e^{-35/1093} = 3.2\%$ which is smaller than the current reported error. The magnetization was calculated as $M_{LS} = \frac{1}{2}\gamma h C N_A P$ where γ is the proton gyromagnetic ratio, h Planck's constant, C the proton concentration, N_A Avogadro's number, and P the liquid-state polarization.

ESR spectrum autocorrelation integral

In thermal mixing the efficiency of the polarization transfer depends on the autocorrelation integral of the radical ESR spectrum evaluated at the Larmor frequency of the nucleus of interest (^{1}H at 285.5 MHz for this study) according to the formula $\int_{-\infty}^{\infty} g(\omega) \cdot g(\omega - \omega_{1H}) d\omega$, where $g(\omega)$ represents the normalized radical spectral function. The autocorrelation integrals of the three samples is reported in Supplementary Figure 2 calculated as follows: PYR_sample = 0.0921; 2CPYR_sample = 0.3449; TEMPOL_sample = 0.4910.

Supplementary References

1. Stoll, S. & Schweiger, A. EasySpin, a comprehensive software package for spectral simulation and analysis in EPR. *J Magn Reson* 178, 42-55, doi:10.1016/j.jmr.2005.08.013 (2006).
2. Capozzi, A. *et al.* Efficient Hyperpolarization of U-(13) C-Glucose Using Narrow-Line UV-Generated Labile Free Radicals. *Angewandte Chemie* 58, 1334- 1339, doi:10.1002/anie.201810522 (2019).
3. Goertz, S. T. *et al.* Highest polarizations in deuterated compounds. *Nucl Instrum Meth A* 526, 43-52, doi:10.1016/j.nima.2004.03.148 (2004).
4. V.V. Krishnan *et al.* Radiation damping in modern NMR experiments: Progress and challenges *Pro.g Nuc.l Magn. Reson. Spectrosc.*68, 41-57, doi:10.1016/j.pnmrs.2012.06.001
